# Transformation of Specific Dispersion Interactions between Cellulose and Polyacrylonitrile in Solutions into Covalent Interactions in Fibers

**DOI:** 10.3390/ma16175843

**Published:** 2023-08-26

**Authors:** Markel I. Vinogradov, Lyudmila K. Golova, Igor S. Makarov, Galina N. Bondarenko, Ivan S. Levin, Natalia A. Arkharova, Valery G. Kulichikhin

**Affiliations:** 1 A.V. Topchiev Institute of Petrochemical Synthesis, Russian Academy of Sciences, 29, Leninsky Prospekt, 119991 Moscow, Russia; m.i.vinogradov1989@yandex.ru (M.I.V.); glk@ips.ac.ru (L.K.G.); bond@ips.ac.ru (G.N.B.); levin@ips.ac.ru (I.S.L.); 2 A.V. Shubnikov Institute of Crystallography, Federal Research Center Crystallography and Photonics, Russian Academy of Sciences, 119333 Moscow, Russia; natalya.arkharova@yandex.ru

**Keywords:** cellulose, polyacrylonitrile, *N*-methylmorpholine-*N*-oxide, dry-jet wet spinning, fibers, structure, crystallinity, mechanical and thermal properties

## Abstract

Morphological transformations in emulsions of cellulose and polyacrylonitrile (PAN) ternary copolymers containing acrylonitrile, methyl acrylate, and methylsulfonate comonomers in *N*-methylmorpholine-*N*-oxide were studied over the entire range of concentrations depending on temperature and intensity of the deformation action. Based on the morphological and rheological features of the system, the temperature-concentration range of spinnability of mixed solutions was determined, and composite fibers were spun. The fibers are characterized by a heterogeneous fibrillar texture. Studies of the structure of the fibers, carried out using X-ray diffraction analysis, revealed a decrease in cellulose crystallinity with an increase in the content of PAN. The study of the thermal properties of the obtained fibers, carried out using DSC, and chemical transformations in them in a wide temperature range by high-temperature diffuse reflection IR spectroscopy made it possible to reveal a new intense exothermic peak on the thermograms at 360 °C, which according to the IR spectra corresponds to the transformation of intermacromolecular physical interactions of the PAN and cellulose into covalent bonds between polymers. In addition, the ester groups found during the thermal treatment of the PAN part of the composite fibers in the pyrolysis zone can have a key effect on the process of their further carbonization.

## 1. Introduction

Among the dual-use fibers that are popular both in the textile and other industries, polyacrylonitrile (PAN) and cellulose fibers are of particular interest due to the fact that they are not only sources of wool-like and cotton-like materials, respectively, but also precursors of carbon fibers (CF) of different assortments [1,2]. CFs from PAN are characterized by high values of carbon yield (up to 50%) and high mechanical characteristics [3,4,5], which render it possible to use them as reinforcing components of composite materials. At the same time, CFs from cellulose with lower values of carbon yield (about 15–20%) and moderate mechanical characteristics, due to high thermal conductivity, are completely indispensable as heat-protection materials and, due to the increased values of the thermal expansion coefficient [6], are well compatible with mineral fibers of the basalt type in materials for thermal protection of nuclear reactors [7].

Let us briefly consider some features of the chemical structure and conditions for processing both polymers into fibers. Their main feature is the non-melting of both PAN homopolymer and cellulose due to strong nitrile-nitrile interactions in the first case and a dense network of hydrogen bonds in the second. To regulate the structure of PAN and its thermal behavior, comonomers are introduced into polymer chains, usually containing ester and acid groups: alkyl acrylates and acids—itaconic, acrylic, etc. [8,9,10]. However, the main method for producing PAN fibers is a solution (wet and dry-jet wet spinning), while the melt spinning method is still under development [11,12,13]. The main difficulties in obtaining melts of PAN copolymers lie in the polarity of the PAN nitrile groups, which have a high dipole moment equal to 3.9 D [14]. The dipole-dipole interactions that arise between polar groups are accompanied by a high melting temperature of the polymer, which is higher than its degradation point [15].

Aprotic organic solvents such as dimethylsulfoxide (DMSO), dimethylformamide (DMF), dimethylacetamide (DMAA), ethylene carbonate, ionic liquids, aqueous solutions of salts such as zinc chloride (ZnCl_2_), sodium thiocyanate (NaSCN), etc. are widely used to obtain PAN spinning solutions [16,17,18]. The content of PAN in dopes, depending on the nature and amount of comonomers and molecular weight of the copolymer, is usually in the range of 12–18% [19]. A further increase in the PAN content in solutions leads to a significant increase in viscosity, which exceeds 10^4^ Pa·s [20,21,22,23].

It was shown for the first time in [24,25] that *N*-methylmorpholine-*N*-oxide (NMMO) has a high dissolving activity not only for hydrophilic polymers such as cellulose, which is widely used in the production of cellulose fibers, but also for hydrophobic PAN. NMMO containing ~8–10% water makes it possible to obtain PAN solutions with concentrations up to 55%. The production of CFs from PAN is associated with a long thermal treatment of precursors (thermooxidative stabilization in the presence of oxygen and carbonization in an inert atmosphere) [26,27]. To increase the efficiency of thermal-oxidative stabilization, modify the properties of the resulting CFs, and replace partly synthetic polymers with renewable natural polymers, such natural polymers as cellulose, lignin, etc. are used as co-components of spinning dispersions [28,29,30].

In addition, nanocrystalline cellulose (NCC) is introduced into PAN via the spinning suspension [31], and only 0.1% of this additive increases the strength of the white fibers by 20% and the elastic modulus by a third. As well, for carbon fibers obtained from these precursors, an increase in mechanical characteristics was observed. The introduction of up to 2% of NCC nanoparticles with a length of 350 nm and a thickness of up to 20 nm into PAN leads to an increase in the strength and elastic modulus of composite precursor fibers by almost 50% compared to the neat fibers [32]. The carbonization of composite precursors also revealed a significant effect of NCC on the strength and elastic modulus of CFs, which almost doubled compared to carbon fibers obtained from PAN precursors. The authors of [33] managed to obtain composite fibers based on PAN with a NCC content of up to 40%. Thermolysis of composite precursors at a temperature of 1000–1400 °C resulted in CFs with a strength of up to 2.3 GPa and an elastic modulus of up to 265 GPa. Unfortunately, the authors of these publications did not analyze the anisotropic elastic properties of graphite crystal inclusions. Such analysis was performed in [34] for prepregs containing CFs carbon fibers from different sources (PAN and pitch) in epoxy matrix, based on the nanoidentation technique. Elastic constants for extension and shear moduli with various orientations of crystallites relative to the long axis of fibers were calculated, which indicate a strong anisotropy of mechanical properties in longitudinal and transversal directions. The presence of two precursor components—CFs and NCC—requires estimating orientation parameters for both of them.

In addition to using nanocrystalline cellulose, ordinary cellulose for chemical processing can also be used as a PAN partner. It is the use of NMMO as a solvent of both polymers that allows for expanding the concentration limits of mixed spinning solutions to create composite CF precursors on their basis. However, it should be borne in mind that cellulose and PAN are thermodynamically incompatible polymers and remain incompatible in a common solvent, NMMO; therefore, all mixed solutions in NMMO are two-phase, i.e., emulsions. Emulsions are movable colloid systems that change morphology under the action of different factors. Especially important is deformation action leading to the transformation of emulsion morphology due to orientation phenomena. Moreover, the heterogeneous morphology of the spinning compositions should lead to a heterogeneous morphology of the spun fibers. In addition, the specific interaction between functional groups of PAN and cellulose in the presence of a very active solvent and high temperatures should be considered.

That is why the following issues are studied in detail in this paper: the morphology of emulsions depending on the composition and intensity of the deformation; the rheological behavior of the obtained emulsions at different temperatures; the optimal temperature-concentration regimes of the dry-jet wet spinning process for composite fibers; the structural features of polymers in solutions and fibers; and a set of fiber properties. Since these fibers are considered potential precursors of CFs, special attention will be paid to the thermal properties and evolution of the chemical composition of polymer components along the temperature scale (until 600 °C).

## 2. Materials and Methods

### 2.1. Materials

To obtain 18% solutions based on PAN and cellulose in NMMO, powdered cellulose (Baikal Pulp and Paper Mill, Baykalsk, Russia) with a polymerization degree of 600, a moisture content of ~8%, a content in the dry state of the α-cellulose of ~92%, a particle size of no more than 250 μm, and a PAN terpolymer of the following composition: acrylonitrile—93.9%, methyl acrylate—5.8%, and methyl ester of sulfonic acid—0.3% (Mw = 85,000 g/mol) with an average particle size of 50 µm (Goodfellow, Huntingdon, UK) were used.

As a solvent for cellulose and PAN, we used NMMO with T_m_ = 120 °C (water content ~10%), manufactured by Demochem (Shanghai, China). To inhibit the thermooxidative degradation of cellulose, 0.5% of propyl gallate (Sigma-Aldrich, St. Louis, MO, USA) was introduced into the system under dissolution.

### 2.2. Methods

#### Preparation of Dopes

At the first stage of the preparation of mixed spinning solutions, solid-phase polymer systems (cellulose-PAN-NMMO) were subjected to preliminary activation under conditions of all-around compression and shear deformation according to the procedure described in [24,35]. To obtain liquid solutions, the activated solid-phase pre-solutions were passed through the operating zone of a HAAKE Minilab II twin-screw laboratory mixer (ThermoFisher Scientific, Dreieich, Germany) at a temperature of 120 °C and a screw rotation speed of 100 rpm.

The viscosity of the solutions was evaluated on a HAAKE MARS 60 Rheometer (ThermoFisher Scientific, Dreieich, Germany) and a Physica MCR 301 (Anton Paar, GmbH, Graz, Austria) rotary rheometer. As operating units, a cone-plane with a diameter of 20 mm and an angle of one degree under steady-state deformation in the shear stress τ range of 10–10^6^ Pa and a cylinder-cylinder unit with an inner diameter of 10 mm were used. To exclude contact of the sample with the environment, the end of the gap was surrounded with PMS-100 silicone oil (Silan, Moscow, Russia). The tests were carried out in the temperature range of 110–130 °C.

The morphology of solutions was studied using polarizing microscopy (Boetius microscope, VEB Kombinat Nadema, Ruhla, Germany, former DDR).

### 2.3. Fiber Spinning

The spinning of composite fibers was carried out by the dry-jet wet method on a Rheoscope 1000 capillary viscometer (CEAST, Torino, Italy) equipped with a winding device at temperatures of 100–140 °C.

### 2.4. Fiber Characterization

#### 2.4.1. Structural and Morphological Characterization

The fiber structure was studied by X-ray diffractometry and IR Fourier spectroscopy. X-ray diffraction patterns were obtained using a Rigaku Rotaflex RU-200 setup (Rigaku Corporation, Tokyo, Japan) equipped with a rotating copper anode (linear focus 0.5–10 mm, source operation mode 50 kV–100 mA, wavelength of characteristic CuKα radiation λ = 1.542 Å, secondary graphite monochromator), a D-Max/B horizontal goniometer, and a scintillation detector. The X-ray survey was carried out in the transmission mode according to the Bragg-Brentano scheme in the continuous θ–2θ scanning mode in the angular range of 5–40°, at a speed of 2°/min with a scanning step of 0.04°. The measurements were carried out at room temperature. The objects used were bundles consisting of 100–150 monofilaments, which were fixed on a flat square aluminum frame perpendicular to the plane of rotation of the detector (equatorial position).

Estimation of crystallinity index *CI* and background removal were performed according to the Segal method [36,37,38]:

*CI* = (*I*_002_ − *I*_*am*_)/*I*_002_ × 100, (1)

where *I*_002_—the intensity of the 002 reflex and *I_am_*—the intensity of diffraction between reflexes of 110 and 200 at 2*θ* = 18°, which corresponds to the amorphous phase of cellulose.

The crystallite size was estimated according to the Scherrer method [39], according to Equation (2):

*D* = 0.89*λ*/*β* cos *θ*, (2)

where *D*—the average crystallite size, *λ*—the X-ray wavelength, *β*—the integral reflection width (in radians), and *θ*—the diffraction angle (Bragg angle).

The morphology of the surface and transverse fiber cleavage was studied by the low-voltage scanning electron microscope FEI Scios (Hillsboro, OR, USA) at an accelerating voltage of less than 1 keV in the secondary electron mode.

The IR spectra of the composite fiber with a composition of 60% cellulose—40% PAN were recorded by high-temperature IR diffuse reflectance spectroscopy in situ (DRIFTS spectra) in the temperature range 25–450 °C in an argon atmosphere on a HYPERION-2000 IR microscope coupled with an IFS IR-Fourier spectrometer −66 v/s Bruker (Billerica, MA, USA) (crystal—Ge, scan 50, resolution 2 cm^−1^, range 600–4000 cm^−1^) (Bruker Optics, Ettlingen, Germany).

#### 2.4.2. Mechanical Testing

The mechanical properties of the fibers were measured on an Instron 1122 tensile machine (Instron, Norwood, MA, USA) at an extension rate of 10 mm/min on a base of 10 mm.

#### 2.4.3. Thermal Characterization

The thermal behavior of the fibers was studied by differential scanning calorimetry (DSC) on a thermal analysis device, TGA/DSC1 Mettler Toledo (Mettler Toledo, Greifensee, Switzerland). The measurements were carried out in aluminum oxide crucibles with a volume of 70 µL in the temperature range of 30 to 400 °C at a heating rate of 10 °C/min. The inert gas (argon) flow rate was 10 cm^3^/min.

## 3. Results and Discussion

**Figure 1:**
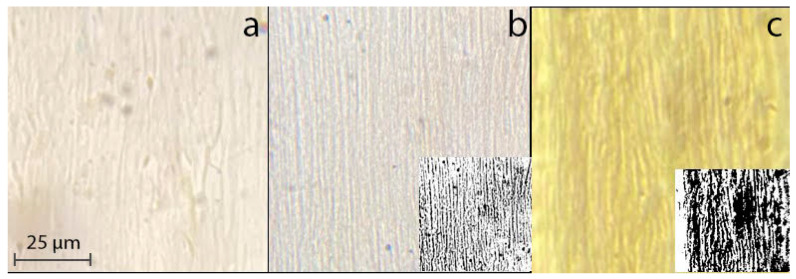
Morphology of 18% mixed solutions containing 30 (**a**), 50 (**b**), and 80% (**c**) PAN solutions, obtained by shear of the emulsion layer.

The morphology of dopes plays an important role in their processing into spun fibers. For two-phase solutions of cellulose and PAN in NMMO, the morphology is determined by the history of preparation, composition, and intensity of the deformation. The intense mixing of the emulsion, which can be achieved in a twin-screw extruder, results in the formation of a micro-heterogeneous dispersion over the entire composition range. However, as can be seen from the micrograph (Figure 1a), the emulsion containing a 30% PAN solution, after flowing through the capillary of the viscometer, is textured, forming fibrils with a diameter of no more than 1 μm, oriented in the flow direction. With an increase in the emulsion of the dispersed phase of the PAN solution up to 50 and more percent, i.e., when the PAN solution becomes the dispersion medium, the fibrillar texture of the emulsion is not only preserved but becomes more regular (Figure 1b,c).

**Figure 2:**
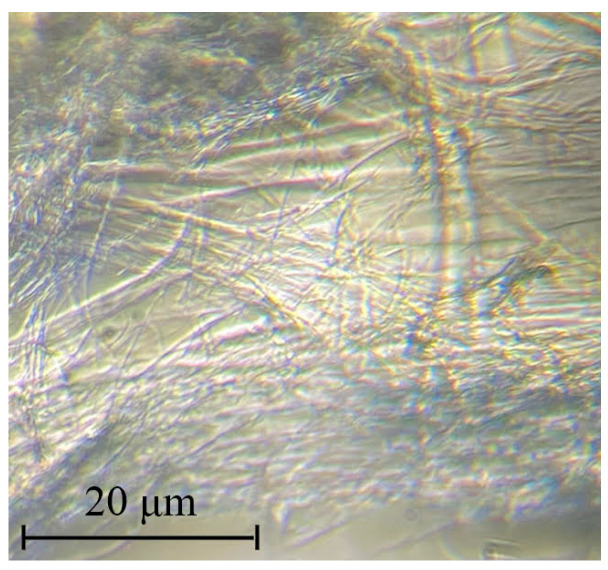
A micrograph of cellulose microfibers after PAN removal from composite fibers that previously contained 60% PAN.

The color processing of photographs makes it possible to more clearly visualize the formed extended fibrils (see inserts in Figure 1b,c). The nature of the observed fibrils was determined by the method of selective dissolution of PAN in DMF. The isolated dispersed phase is, as can be seen from Figure 2, long, thin cellulose microfibers with a diameter of about 1–2 μm.

Thus, when the content of the dispersed phase of the PAN solution in the composite emulsion is around or more than 30%, under shear conditions, the dispersed droplet-like morphology of the system transforms to the fibrillar one.

**Figure 3:**
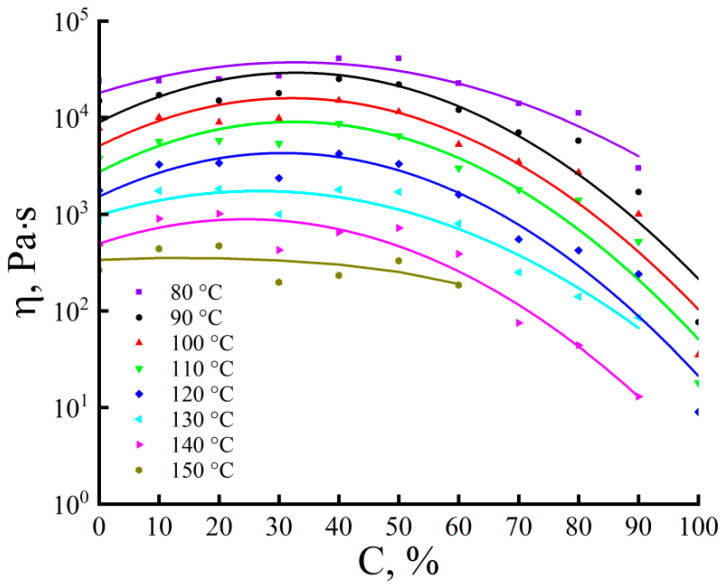
Dependences of the viscosity of 18% mixed solutions (at a shear rate of 0.01 s^−1^) on the content of the PAN solution.

To reveal the influence of the morphological features of the studied emulsions on the nature of their flow, the rheological behavior of the studied emulsions of different compositions was studied in a wide temperature range (80–150 °C). The shape of all dependencies of viscosity on PAN content (Figure 3) is almost identical at all temperatures, and the differences are only in the absolute values of viscosity, which are predetermined by the emulsion composition and temperature. The introduction of PAN into the cellulose solution leads to some increase in the viscosity of the emulsions compared to the cellulose solution up to a 50% PAN content, i.e., to the point of phase inversion. For all solutions, there is a positive deviation from the dependence predicted by the rule of logarithmic viscosity additivity for a two-component system.

**Figure 4:**
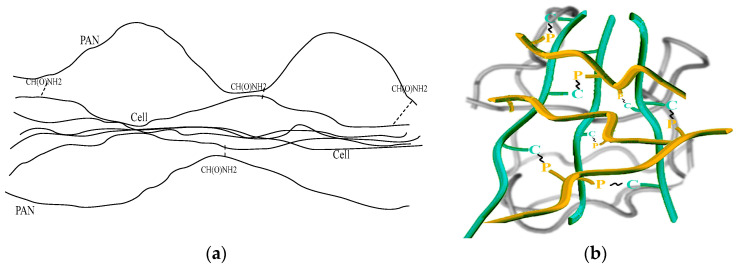
Schematic image of PAN (P)-cellulose (C) associate in mixed solution (**a**) and illustration of specific H-bond formation between polymers via –C-C(O)NH_2_ (**~**) links (**b**).

This means a strong intermolecular interaction between the polymer components of the system. To analyze its mechanism, it makes sense to return to earlier works devoted to this issue [40,41]. Thus, IR spectral studies have shown that in the process of dissolution and fiber formation in NMMO at 120 °C, PAN, except for the partial cyclization of nitrile groups, undergoes deeper chemical transformations, namely hydrolysis reactions with the formation of acrylamide units—C-C(O)-NH_2_, which easily (with the participation of water molecules or directly) form H-bonds with cellulose hydroxyl groups. The appearance of sufficiently strong contacts between the chains of PAN and cellulose leads to a change in the conformation of PAN and cellulose macromolecules, and their regions, not connected by hydrogen and dispersion bonds, will repel each other due to the hydrophobicity of the preserved nitrile groups. In other words, cellulose and PAN macromolecules form unique associates within which cellulose chains are located and surrounded by PAN macromolecules (see schemes in Figure 4), i.e., the mixed PAN and cellulose emulsion consists of self-formed macromolecular formations with more strong intra-associate interactions and weaker inter-associate interactions.

It is for this reason that fibrils appear in the emulsion during the flow, which are caused not by the deformation of the droplets of the disperse phase but by the specific intra- and intermacromolecular interaction between polymers, which enhances the longitudinal and weakens the transverse ordering of macromolecules. The implementation of such associates in the system can lead to an increase in viscosity. A further increase in the PAN content in the system is above 50%, i.e., when the phase inversion point is overcome and the low-viscous PAN solution phase becomes a dispersion medium, the viscosity of emulsions begins to decrease.

Previous experience has shown that for the successful spinning of composite fibers, the viscosity of the spinning dope must be in the range between 10^3^ and 10^4^ Pa·s, which corresponds to emulsions containing up to 60% PAN in the temperature range of 100 to 130 °C. These temperatures were chosen for spinning fibers according to the traditional dry-jet-wet scheme. The hot jet of emulsion emerging from the capillary was drawn into the air gap with a drawing ratio of up to 25 at a spinning rate of up to 100 m/min. The distance between the spinneret and the surface of the coagulation bath was 10 cm. Next, the solution jet entered the water bath at room temperature (T = 20 ± 2 °C). The path in the bath did not exceed 5 cm. Moreover, the as-spun fiber was directed to the winding device.

To completely remove NMMO from the as-spun fiber, it was washed with water at room temperature for a day in a static mode, and after this operation, the fiber was dried in a free state. The low viscosity of 18% PAN solutions in NMMO did not allow the formation of fibers even at a significant decrease in temperature. That is why PAN fibers were spun from 30% solutions, for which at 120 °C the viscosity value exceeds 10^2^ Pa·s.

**Figure 5:**
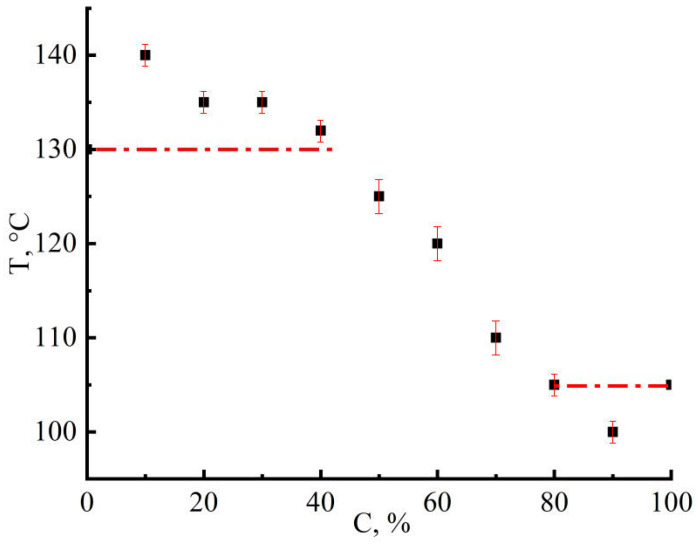
The temperature-concentration window of the stable composite fibers spinning. Dashed lines describe the limits of successful spinning according to rheological data.

The study of the spinning process of emulsions of different phase compositions made it possible to determine the temperature-concentration range of stable fiber formation. Figure 5 shows the dependence of stable fiber spinning temperatures on the content of the PAN solution in the emulsion.

The plotted concentration dependence has two limiting regions corresponding to the temperatures of stable formation of 18% cellulose solutions (130 °C) and 30% PAN solutions (105 °C) in NMMO. The conditions revealed for the stable formation of the studied emulsions are directly related to their morphological and rheological features.

**Figure 6:**
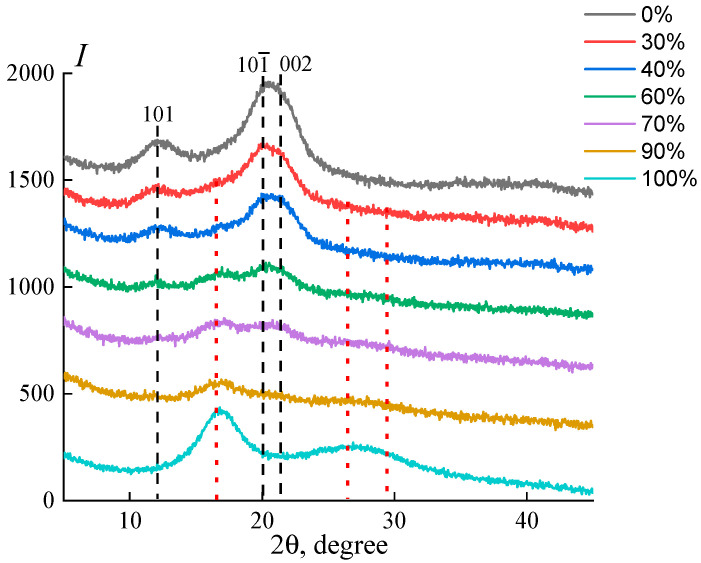
Equatorial diffraction patterns of composite fibers formed from 18% mixed solutions with different PAN contents (indicated in the graph).

The obtained composite fibers were investigated by various methods. First of all, their structure was studied by X-ray diffraction analysis, and the corresponding diffractograms are shown in Figure 6.

For cellulose fibers, the main reflections are in the angular positions 2*θ* ~ 12.1°, ~20.1°, and ~21.5°, which correspond to the crystallographic planes (101), ($10\bar{1}$), and (002) (marked in Figure 6 by a black dotted line), which are inherent to regenerated cellulose [42]. The diffraction pattern of PAN fibers contains two reflections at 2*θ* = 16.9° (d = 0.524 nm) and 2*θ* = 29.4° (d = 0.303 nm), as well as a wide reflection in the region of 25.7° [41], marked in a red dotted line. Regarding the structure of PAN, two hypotheses are considered in the literature. According to the first one, reflections 1 and 3 in the diffraction patterns are attributed to the crystalline phase of PAN and the halo-like peak 2 (2*θ* ~ 26°) to the amorphous phase [43]. According to the second point of view, reflection 2 in the diffraction pattern is assigned to a rotationally disordered mesophase [44].

All the diffraction patterns presented in Figure 6 of the composite fibers are a superposition of the diffraction patterns of both polymers, and the characteristic PAN reflections begin to appear in the diffraction patterns of composite fibers only at concentrations above 30%. Hence, on the diffraction pattern of fibers composed of 60% cellulose and 40% PAN, only the PAN reflection is observed in the region of 2*θ* ~ 16.9°, and the other two reflections at 2*θ* = 29.4° and 25.7° are absent (or have very low intensities). The main reflections of cellulose also decrease with an increase in the proportion of PAN in fiber. At the same time, the ratio of the intensities of the main reflections also decreases, which indicates a decrease in the ordering of the cellulose structure in the (101), ($10\bar{1}$), and (002) planes. With an increase in the PAN content to 70%, the cellulose reflections practically disappear, and the first PAN reflection, on the contrary, increases, while other characteristic PAN reflections appear as a wide, amorphous halo. When the PAN content reaches 90%, the cellulose reflection at 2*θ* ~ 12.1° completely disappears, and the other two are hidden under the right shoulder of the first PAN reflection.

**Figure 7:**
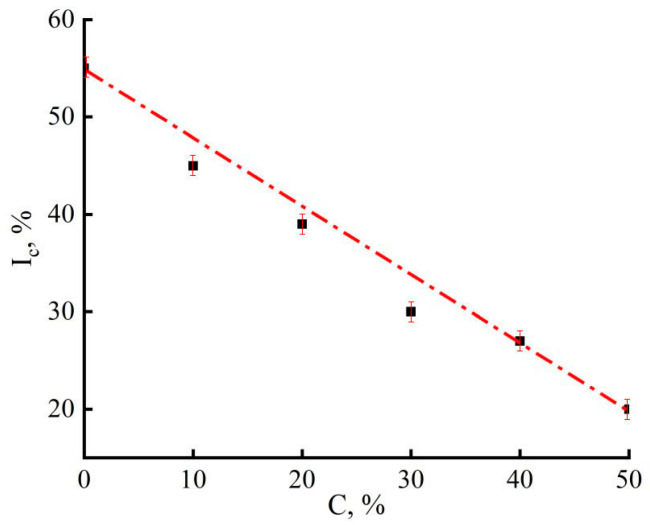
Dependence of the cellulose crystallinity index in composite fibers on the PAN content.

Figure 7 shows the dependence of the cellulose crystallinity index on the PAN content in composite fibers.

**Figure 8:**
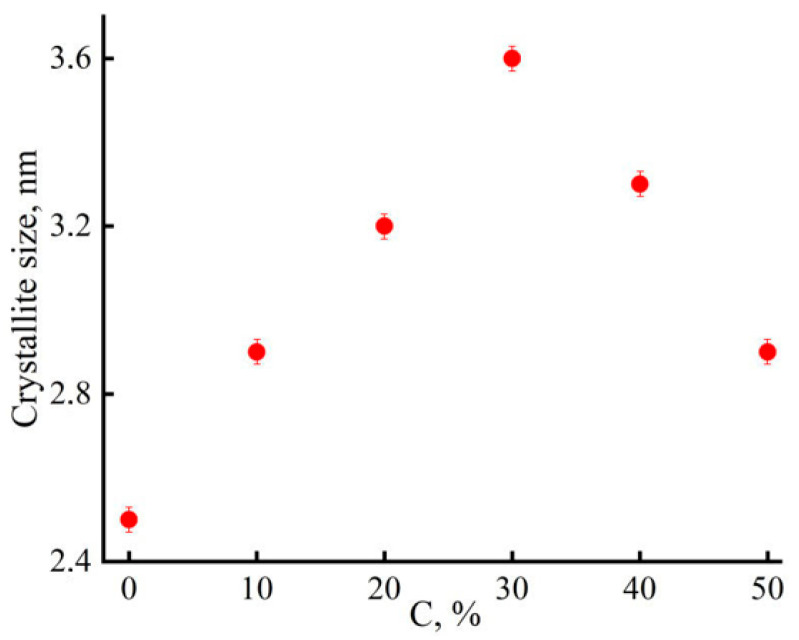
Dependence of the averaged crystallite sizes on the content of PAN in the system.

As is seen from the above dependence, the introduction of PAN into cellulose leads to a gradual decrease in the degree of crystallinity and, accordingly, to cellulose amorphization. According to the nature of the change in the obtained equatorial diffraction patterns, the introduction of cellulose into PAN also disturbs the regularity of the ordering of PAN macromolecules. Thus, the combination of these two polymers in the composition leads to a disturbing effect in terms of the manifestation of their structural order. At the same time, oddly enough, the size of cellulose crystallites, estimated by the Scherrer equation for the reflection corresponding to the ($10\bar{1}$) plane, has an extremal dependence on the composition (Figure 8).

Apparently, this is due to the above-discussed morphology of composite fibers caused by strong intermacromolecular interaction, which is, in fact, a gradient, i.e., more pronounced in the longitudinal direction, along the long axes of macromolecular associates. However, this effect manifests itself only up to a PAN concentration in the fiber of the order of 30% or more. Further, the phenomenon of inhibition of crystallization processes in each of the polymers comes into force as a result of blocking their individuality and a decrease in the size of the crystallites in the cellulose phase. This is a fundamental difference from the structure of Orcel cellulose fibers [45], in which, due to the high orientation of amorphous regions, a conditional increase in the degree of crystallinity and crystallite size in the longitudinal direction is observed.

**Figure 9:**
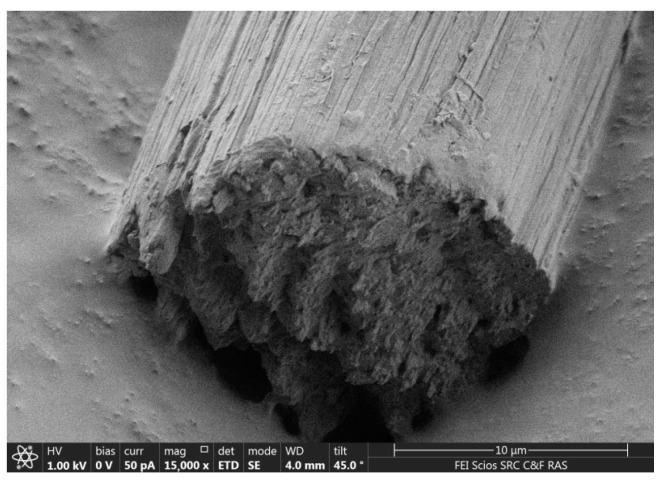
Micrograph of a composite fiber of composition: 40% cellulose—60% PAN.

The morphology of the composite fiber with a high PAN content is shown in Figure 9.

Unlike cellulose fibers such as Lyocell or Orcel, in which the high longitudinal orientation of both crystalline and amorphous regions promotes the formation of fibrils with a size not exceeding 0.08–0.1 μm with such dense packing that they do not appear on the cross sections of the fibers, the obtained composite fibers are characterized by a larger heterogeneous texture, formed by microfibers of the order of 1 μm rather than by fibrils. Microfibers are also heterogeneous and consist of many oriented fibrillar subunits. The surface of the fiber is striated due to the microfibers protruding from the surface, but the cross-section is generally round.

It is difficult to state unambiguously the phase compositions of the resulting structures, but the cellulose fibrils isolated after the removal of PAN suggest that PAN macromolecules or their associates are located on the surface of cellulose formations [40]. By the way, the high interfacial forces exerted between non-lignified fibers (which are rich in cellulose content) and polar matrixes were observed in [46]. Simultaneously, it is possible to consider that a certain contribution to the structure formation of the composite fiber is also made by different rates of mass transfer processes of polymer solutions of the emulsion in contact with the coagulant, which leads to high fiber heterogeneity.

**Figure 10:**
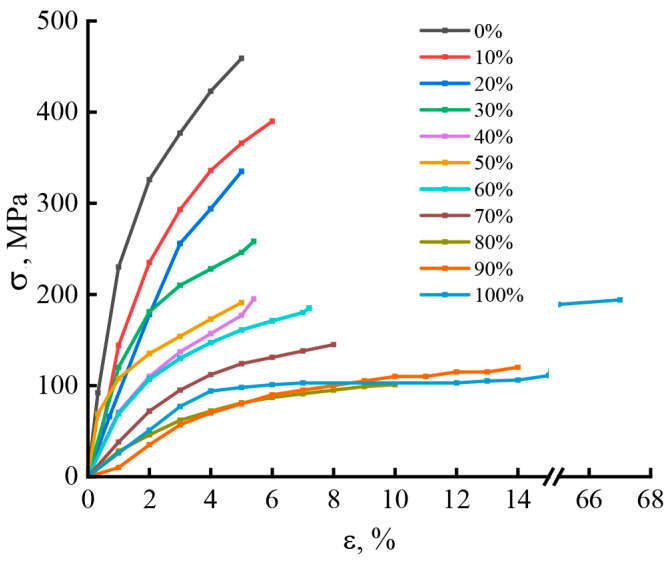
Stress versus strain for all composite fibers.

The lack of monolithic character in the composite fiber affects its mechanical characteristics. As is seen from Figure 10, where stress-strain curves are presented, the introduction of PAN into cellulose leads to a decrease in the strength and modulus of composite fibers while maintaining low values of elongation at break, which are intrinsic for cellulose fibers.

Comparison of the deformation curves of fibers shows that the introduction of the PAN copolymer into the cellulose matrix leads to a decrease in the ability to resist fracture, but the ability to deform under the influence of mechanical stresses gradually increases and reaches the maximum values for PAN fibers. The character of fracture changes from brittle to ductile at a PAN concentration in the composite fiber of the order of 70–80%. Such a clear deterioration in the mechanical properties of composite fibers with the accumulation of PAN in their composition is apparently associated with a specific interaction between the functional groups of polymers, which leads to the loss of each of the polymers’ individuality.

**Figure 11:**
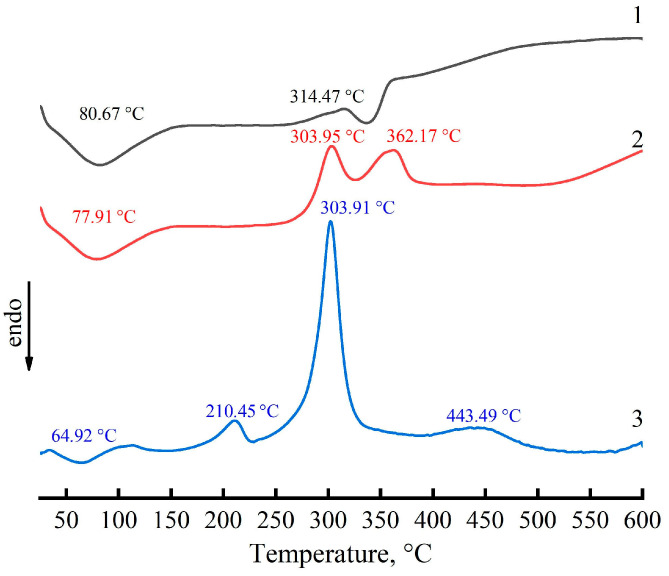
DSC thermograms of cellulose (1), composite (60% cellulose—40% PAN) (2), and PAN fibers (3).

In particular, as far as such losses occur deep and as far as it is important from the standpoint of using them as precursors of carbon products, Figure 11 reveals DSC thermograms of cellulose, PAN, and composite fibers of composition: 60% cellulose—40% PAN.

The thermogram of cellulose fiber is characterized by the presence of an endothermic peak with a maximum of 80.7 °C and an extended exothermic peak at 314 °C. The endothermic peak in the given thermogram corresponds to the removal of adsorption water, and the exothermic peak at 314 °C corresponds to the first stages of pyrolysis. The thermogram of PAN fibers contains three exothermic peaks: the peak at 215 °C can be due to PAN crystallization or mesophase ordering; the peak at 303 °C is due to the cyclization of nitrile groups; and the high-temperature peak corresponds to deeper transformations of PAN or its decomposition products under pyrolysis conditions.

**Figure 12:**
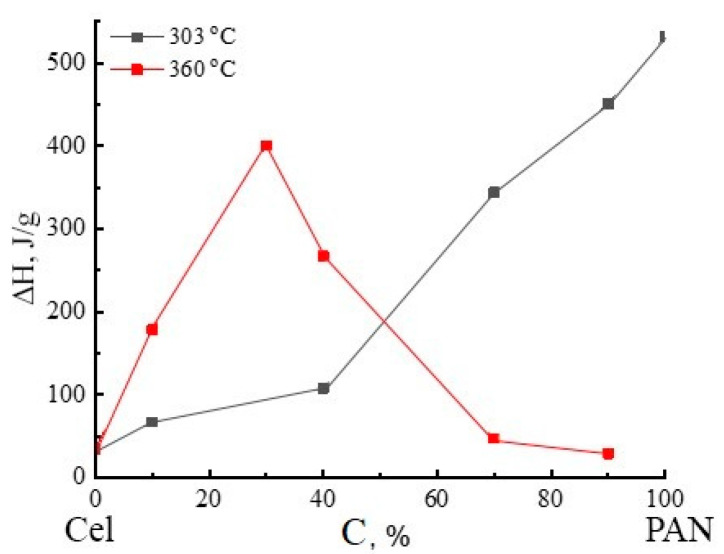
Dependences of the latent heats of exothermic peaks at 303 and 360 °C on the composition of mixed fibers.

The composite fiber thermogram is not a superposition of the thermograms of two initial polymers. Of particular interest is the appearance of a new, rather intense exothermic peak in the region of 360 °C and its nature. Certain considerations about the reasons for the appearance of this peak can be made by analyzing the change in the heats of two exothermic peaks at 303 and 360 °C, depending on the composition of composite fibers (Figure 12). As can be seen from the figure, the heat of the new peak at 360 °C increases up to 30% PAN content in the mixture system and then decreases and practically disappears as the system approaches 100% PAN, while the thermal effect of the PAN cyclization reaction at 303 °C increases monotonically as the PAN content in the system increases.

As can be seen from the date presented in Figure 12, the thermal effect of structural-chemical transformations of cellulose at the stage of pyrolysis is more than an order of magnitude less than the thermal effect of PAN cyclization, while the positions of the maxima of the exothermic peaks of cellulose and PAN are close and correspond to 314 and 303 °C, respectively. It is impossible to draw any conclusions from the thermogram of the composite fiber about the nature of cellulose transformations during heat treatment; therefore, the features of cellulose structural rearrangements were established based on the analysis of IR spectra obtained in the temperature range of 25–400 °C, i.e., in the area of thermal oxidation of PAN in the air atmosphere.

**Figure 13:**
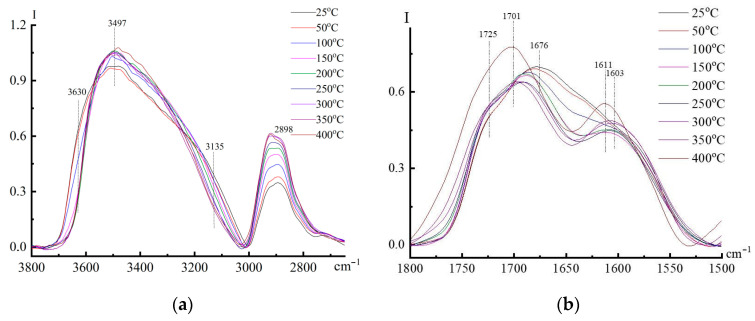
DRIFTS spectra of 60% cellulose—40% PAN composite, recorded at different temperatures in the absorption regions of OH, N-H, C-H (**a**) and C=O, C=C (**b**) bonds.

First of all, a feature of the spectra is the disappearance at certain temperatures of the absorption bands recorded for the neat samples and the appearance of new groups. Hence, in the region of absorption of OH, N-H, and C-H in PAN (Figure 13a), at temperatures above 200 °C, the long-wavelength shoulder of the 3135 cm^−1^ band disappears, which may be due to the disappearance of associated -N-H…O links since the bands from them usually lie at 3200 cm^−1^ and below.

The disappearance of amide groups from PAN was accompanied by a change in the location of C=O groups (Figure 13b). It can be seen that in the temperature range of 25–200 °C, the intensity of the band at 1676 cm^−1^, which is for C=O valence bonds in amide groups, drops sharply, and at 200 °C, it completely disappears. Instead of this band, a new intense band appears at 1701 cm^−1^ from C=O stretching vibrations, intrinsic already for the carboxyl group –C(O)OH, and its shoulder is 1725 cm^−1^ for ester groups. This means that during the heat treatment of fibers, water is released; that is, it is easy to imagine the hydrolysis of amide groups with the formation of carboxyl groups:

RC(O)NH_2_ + H_2_O → RC(O)OH + NH_3_

The ester groups can easily be formed from associates of amide groups with OH groups of cellulose:

R_1_C(O)N(H)H…O(H)R_1_ → R_1_C(O)OR_2_ + NH_3,_

where R_1_ is the PAN chain and R_2_ is the cellulose chain.

Hence, it can be concluded that non-covalent specific interactions of PAN and cellulose through hydrogen bonds formed during the dissolution of polymers in NMMO and the formation of the composite fiber, upon further thermal treatment, are transformed into covalent bonds between polymers, and the main role in the formation of such bonds belongs to amide groups.

It is extremely important to note that the ester groups formed during heat treatment, both in PAN and in cellulose, introduce certain changes in the conformations of polymer macromolecules, thereby favoring the formation of carbon-carbon bonds with further transformation into carbocyclic compounds, i.e., having a kind of catalytic effect on the carbonization process. The formation of carbonyl and carboxyl groups in the composite precursor of cellulose with PAN during in situ pyrolysis can be an important positive factor in the process of obtaining CFs from them.

**Figure 14:**
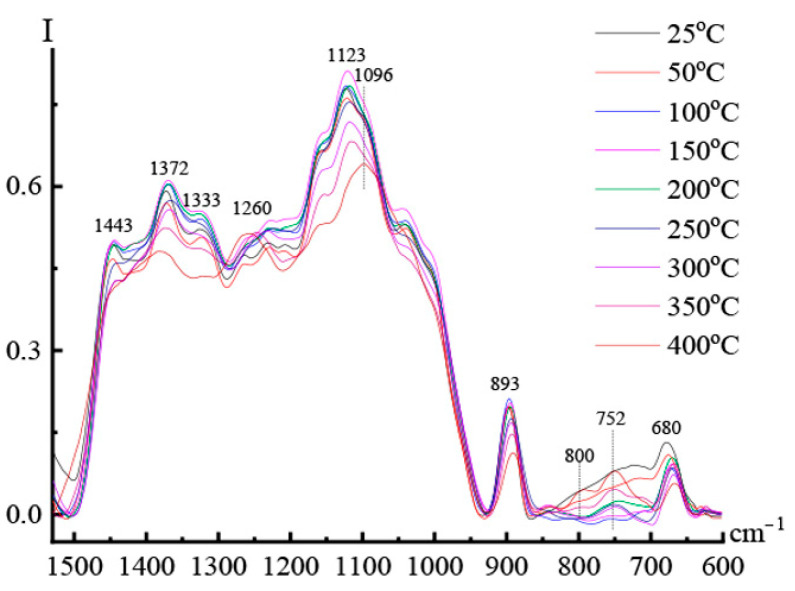
The DRIFTS spectra of a composite fiber composed of 60% cellulose—40% PAN were recorded in the temperature range 25–400 °C in the absorption region of C-O bonds.

In Figure 14, spectra allow us to analyze what happens to cellulose during high-temperature processing of a composite fiber with PAN.

**Figure 15:**
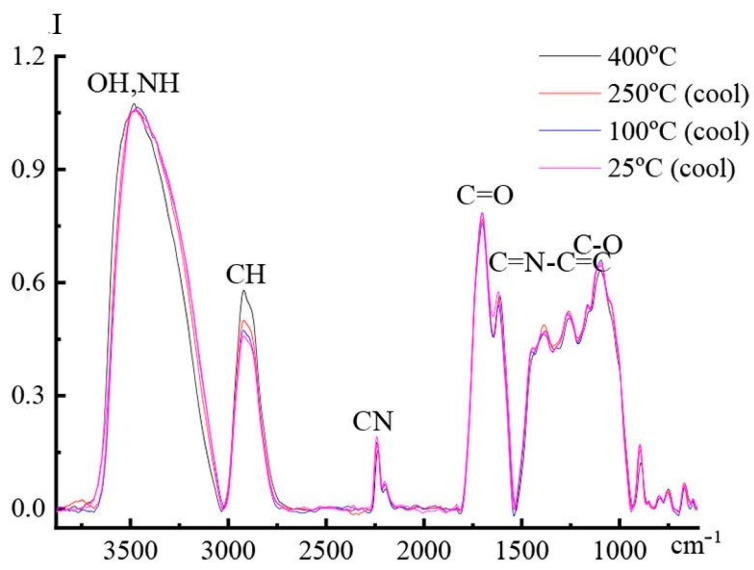
Comparison of the survey DRIFTS spectra of 60% cellulose—40% PAN composite fiber recorded during cooling in argon in the temperature range of 25–400 °C.

The intense band at 1123 cm^−1^ from vibrations of C-OH bonds in glucopyranose cycles is slightly shifted to the region of long waves and changes intensity in a complicated way in the temperature range of 25–250 °C; at higher temperatures, it significantly decreases in intensity. A comparison of the spectra of the composition at 25 °C before heating and after cooling, shown in Figure 15, shows that after heat treatment, 82% of “free” glucopyranose rings remain in the system, and 18% are chemically bound to PAN. The formation of chemical interactions between cellulose and PAN also manifests itself in the spectral bands responsible for the degree of ordering of cellulose. The broad band with a maximum at 680 cm^−1^, which reflects complex vibrations in the ordered regions of cellulose, sharply decreases in intensity with increasing temperature. In the spectra recorded at 400 °C, the bands at 1443 and 1372 cm^−1^, which are considered to be responsible for vibrations of C-C-O and C-O-C bonds in glucopyranose cycles in the crystalline phase of cellulose, disappear completely.

The band at 893 cm^−1^, which is responsible for the amorphous phase of cellulose, also decreases in intensity by 20% after heating the sample compared to the spectrum of the neat mixture. While an assessment of the relative intensity of the band of nitrile groups at 400 °C indicates the transformation of 64% of nitrile groups into other structural forms, only about 20% of cellulose is connected by covalent bonds with PAN. Based on the structure of composite fibers consisting of cellulose fibrils surrounded by PAN macromolecules, it is possible to imagine that covalent bonds concern mainly the surface of cellulose fibrils “protected” by PAN macromolecules. It is a reason for the preservation of 82% of cellulose cycles while more than 60% of nitrile groups in PAN have undergone chemical changes.

The results obtained allow us to conclude that the intermolecular covalent bonds between polymers formed during high-temperature treatment of a composite cellulose-PAN fiber up to 400 °C change the mechanism of cyclization of nitrile groups and slow down the processes of high-temperature structural-chemical transformations of cellulose. Accepting that successful carbonization of the composite precursors depends on the orientation of two neat species: cellulose and PAN, the approach disclosed in [34] for CFs on anisotropic elastic constants could be spread at carbonization on both carbon and graphite inclusions owned by two components.

## 4. Conclusions

1. The morphology of cellulose and PAN emulsions in NMMO with an increase in the PAN content above 30% transforms from finely dispersed droplets to fibrillar, predetermining the rheological behavior of emulsions and the structure of the fibers obtained on their basis.
2. Fibrillation is caused by the specific structure of emulsion, consisting of associates with the inner part of cellulose molecules surrounded by PAN molecules manifesting the hydrophobic repulsion of nitrile groups.
3. The non-covalent interaction in mixed solutions in NMMO at high temperatures consists of H-bonding between amide groups, formed at the cyclization of nitrile links in PAN, and hydroxyl groups of cellulose.
4. The concentration dependence of the optimal temperature for stable fiber spinning is constructed; the composite fibers have been obtained in a wide range of component ratios, and their mechanical properties have been analyzed.
5. During the high-temperature treatment of composite fibers up to 400 °C, the specific dispersion interactions of PAN and cellulose, which are formed during the dissolution of polymers in NMMO and the fibers spinning, are transformed into covalent bonds. This is the first example of PAN-cellulose copolymer synthesis by means of sequential transformation and dispersion interaction into covalent bonds.
6. The driving force of this transformation is the more deep cyclization of nitrile groups in solution of NMMO compared with traditional stabilization of PAN fibers.

## Data Availability

Not applicable.

## References

[1] Geller B.E. (2002). Status and Prospects for Development of Polyacrylonitrile Fibre Production. A Review. Fibre Chem..

[2] Skvortsov I.Y., Maksimov N.M., Kuzin M.S., Toms R.V., Varfolomeeva L.A., Chernikova E.V., Kulichikhin V.G. (2023). Influence of Alkyl Acrylate Nature on Rheological Properties of Polyacrylonitrile Terpolymers Solutions, Spinnability and Mechanical Characteristics of Fibers. Materials.

[3] Kaur J., Millington K., Smith S. (2016). Producing high-quality precursor polymer and fibers to achieve theoretical strength in carbon fibers: A review. J. Appl. Polym. Sci..

[4] Bhat G. (2016). Structure and Properties of High-Performance Fibers.

[5] Der A., Dilger N., Kaluza A., Creighton C., Kara S., Varley R., Herrmann C., Thiede S. (2021). Modelling and analysis of the energy intensity in polyacrylonitrile (PAN) precursor and carbon fibre manufacturing. J. Clean. Prod..

[6] Pradere C., Sauder C. (2008). Transverse and longitudinal coefficient of thermal expansion of carbon fibers at high temperatures (300–2500 K). Carbon.

[7] Dhand V., Mittal G., Rhee K.Y., Park S.J., Hui D. (2015). A short review on basalt fiber reinforced polymer composites. Compos. B Eng..

[8] Fu Z., Gui Y., Liu S., Wang Z., Liu B., Cao C., Zhang H. (2014). Effects of an itaconic acid comonomer on the structural evolution and thermal behaviors of polyacrylonitrile used for polyacrylonitrile-based carbon fibers. J. Appl. Polym. Sci..

[9] Devasia R., Reghunadhan N.C.P., Sadhana R., Babu N.S., Ninan K.N. (2006). Fourier transform infrared and wide-angle X-ray diffraction studies of the thermal cyclization reactions of high molar mass poly(acrylonitrile-co-itaconic acid). J. Appl. Polym. Sci..

[10] Farsani R.E., Raissi S., Shokuhfar A., Sedghi A. (2009). FT-IR study of stabilized PAN fibers for fabrication of carbon fibers. World Acad. Sci. Eng. Technol..

[11] Gupta B.S., Afshari M., Bunsell A.R. (2018). Polyacrylonitrile fibers. Handbook of Properties of Textile and Technical Fibres.

[12] Zeng X., Chen J., Zhao J., Wu C., Pan D., Pan N. (2009). Investigation the jet stretch in PAN fiber dry-jet wet spinning for PAN-DMSO-H_2_O system. J. Appl. Polym. Sci..

[13] König S., Kreis P., Herbert C., Wego A., Steinmann M., Wang D., Frank E., Buchmeiser M.R. (2020). Melt-Spinning of an Intrinsically Flame-Retardant Polyacrylonitrile Copolymer. Materials.

[14] Wu X., Chu Y., Liu R., Katz H.E., Huang J. (2017). Pursuing Polymer Dielectric Interfacial Effect in Organic Transistors for Photosensing Performance Optimization. Adv. Sci..

[15] Lachat V., Varshney V., Dhinojwala A., Yeganeh M.S. (2009). Molecular Origin of Solvent Resistance of Polyacrylonitrile. Macromolecules.

[16] Konkin A.A. (1974). Carbon and Other Heat–Resistant Fiber Materials.

[17] Wu Q.Y., Chen X.N., Wan L.S., Xu Z.K. (2012). Interactions between polyacrylonitrile and solvents: Density functional theory study and two-dimensional infrared correlation analysis. J. Phys. Chem. B.

[18] Ziabicki A. (1976). Fundamentals of Fibre Formation: The Science of Fibre Spinning and Drawing.

[19] Tsai H.A., Chen Y.L., Lee K.R., Lai J.Y. (2012). Preparation of heat-treated PAN hollow fiber membranes for pervaporation of NMP/H_2_O mixtures. Sep. Purif. Technol..

[20] Iovleva M., Smirnova V., Budnitskii G. (2001). The solubility of polyacryonitrile. Fibre Chem..

[21] Jin X., Li L., Xu R., Liu Q., Ding L., Pan Y., Wang C., Hung W., Lee K., Wang T. (2018). Effects of Thermal Cross-Linking on the Structure and Property of Asymmetric Membrane Prepared from the Polyacrylonitrile. Polymers.

[22] Xu L., Qiu F. (2015). Unusual viscosity behavior of polyacrylonitrile in NaSCN aqueous solutions. Polymer.

[23] Byrne N., Leblais A., Fox B. (2014). Preparation of polyacrylonitrile–natural polymer composite precursors for carbon fiber using ionic liquid co solvent solutions. J. Mater. Chem. A.

[24] Makarov I.S., Golova L.K., Kuznetsova L.K., Shlyakhtin A.V., Nifant’ev I.E., Kulichikhin V.G. (2013). Method for Obtaining a Solution of Acrylonitrile-Based Copolymer in *N*-methylmorpholine-*N*-oxide. Patent.

[25] Zeng T., Ke X., Li L., Cheng X., Ni Y., Ouyang X., Zhang X., Chen L., Huang L., Hu H.C. (2020). Quantification of *N*-methyl morpholine *N*-oxide in biorefinery process solution by headspace gas chromatography. Cellulose.

[26] Thunemann A.F. (2000). Dielectric relaxation of polyacrylonitrile in its pristine and cyclized stage. Macromolecules.

[27] Xue Y., Liu J., Liang J.Y. (2013). Correlative study of critical reactions in polyacrylonitrile based carbon fiber precursors during thermal-oxidative stabilization. Polym. Degrad. Stabil..

[28] Chang H., Chien A.T., Liu H.C., Wang P.H., Newcomb B.A., Kumar S. (2015). Gel Spinning of Polyacrylonitrile/Cellulose Nanocrystal Composite Fibers. ACS Biomater. Sci. Eng..

[29] Seydibeyoglu M.O. (2012). A Novel Partially Biobased PAN-Lignin Blend as a Potential Carbon Fiber Precursor. J. Biomed. Biotechnol..

[30] Zhang X., Qi Y., Yang J., Dong S., Liu J., Li J., Shi K. (2021). Insight into stabilization behaviors of Lignin/PAN-derived electrospun precursor fibers. Polym. Degrad. Stabil..

[31] Jiang E., Maghe M., Zohdi N., Amiralian N., Naebe M., Laycock B., Fox B.L., Martin D.J., Annamalai P.K. (2019). Influence of Different Nanocellulose Additives on Processing and Performance of PAN-Based Carbon Fibers. ACS Omega.

[32] Park S.H., Lee S.G., Kim S.H. (2013). The use of a nanocellulose-reinforced polyacrylonitrile precursor for the production of carbon fibers. J. Mater. Sci..

[33] Chang H., Luo J., Liu H.C., Zhang S., Park J.G., Liang R., Kumar S. (2019). Carbon fibers from polyacrylonitrile/cellulose nanocrystal nanocomposite fibers. Carbon.

[34] Shirasu K., Goto K., Naito K. (2020). Microstructure-elastic property relationships in carbon fibers: A nanoindentation study. Compos. B Eng..

[35] Golova L.K., Romanov V.V., Lunina O.B., Platonov V.A., Papkov S.P., Khorozova O.D., Yakshin V.V., Belasheva T.P., Sokira A.N. (1991). Method for Obtaining a Solution for Spinning Fibers. Patent.

[36] Fink H.P., Hofmann D., Philipp B. (1995). Some aspects of lateral chain order in cellulosics from X-ray scattering. Cellulose.

[37] French A.D. (2020). Increment in evolution of cellulose crystallinity analysis. Cellulose.

[38] French A.D., Cintron S.M. (2013). Cellulose polymorphy, crystallite size, and the segal crystallinity index. Cellulose.

[39] Gindl W., Keckes J. (2005). All-cellulose nanocomposite. Polymer.

[40] Vinogradov M.I., Makarov I.S., Golova L.K., Bondarenko G.N., Kulichikhin V.G. (2023). Structural–Morphological and Rheological Features of Joint Solutions of Cellulose and PAN Copolymer in *N*-Methylmorpholine-*N*-Oxide. Polym. Sci. Ser. A.

[41] Makarov I.S., Golova L.K., Vinogradov M.I., Levin I.S., Sorokin S.E. (2019). Structure of Polyacrylonitrile Fibers Produced from *N*-Methylmorpholine-*N*-Oxide Solutions. Fibre Chem..

[42] Kaplan D.L. (2013). Biopolymers from Renewable Resources.

[43] Pawde S.M., Deshmukh K. (2008). Influence of γ irradiation on the properties of polyacrylonitrile films. J. Appl. Polymer Sci..

[44] Liu X.D., Ruland W. (1993). X-ray studies on the structure of polyacrylonitrile fibers. Macromolecules.

[45] Golova L.K., Vasilyeva N.V., Borodina O.E., Krylova T.B., Kuznetsova L.K., Rogovina S.Z., Zelenetsky S.N. (1994). Method of preparing cellulose solution for fabricating shaped articles. Patent.

[46] Marcuello C., Chabbert B., Berzin F., Bercu B.N., Molinari M., Aguié-Béghin V. (2023). Influence of Surface Chemistry of Fiber and Lignocellulosic Materials on Adhesion Properties with Polybutylene Succinate at Nanoscale. Materials.

